# Relevant phosphoproteomic and mass spectrometry: approaches useful in clinical research

**DOI:** 10.1186/2001-1326-1-2

**Published:** 2012-03-29

**Authors:** Elena López, Sarbelio Rodríguez Muñoz, Juan López Pascual, Luis Madero

**Affiliations:** 1 Hospital Universitario Infantil Niño Jesús, Av. Menéndez Pelayo 65, 28009 Madrid, Spain; 2 Hospital Universitario 12 de Octubre, Av. De Córdoba s/n, 28040 Madrid, Spain; 3 Facultad de Medicina de la Universidad Autónoma de Madrid, C/del Arzobispo Morcillo, 428029, Spain

**Keywords:** Phosphoproteomics, Mass spectrometry, Clinical research

## Abstract

**Background:**

"It's not what we do, it's the way that we do it". Never has this maxim been truer in proteomics than now. Mass Spectrometry-based proteomics/phosphoproteomics tools are critical to understand the structure and dynamics (spatial and temporal) of signalling that engages and migrates through the entire proteome. Approaches such as affinity purification followed by Mass Spectrometry (MS) have been used to elucidate relevant biological questions disease *vs*. health. Thousands of proteins interact via physical and chemical association. Moreover, certain proteins can covalently modify other proteins post-translationally. These post-translational modifications (PTMs) ultimately give rise to the emergent functions of cells in sequence, space and time.

**Findings:**

Understanding the functions of phosphorylated proteins thus requires one to study proteomes as linked-systems rather than collections of individual protein molecules. Indeed, the interacting proteome or protein-network knowledge has recently received much attention, as network-systems (signalling pathways) are effective snapshots in time, of the proteome as a whole. MS approaches are clearly essential, in spite of the difficulties of some low abundance proteins for future clinical advances.

**Conclusion:**

Clinical proteomics-MS has come a long way in the past decade in terms of technology/platform development, protein chemistry, and together with bioinformatics and other OMICS tools to identify molecular signatures of diseases based on protein pathways and signalling cascades. Hence, there is great promise for disease diagnosis, prognosis, and prediction of therapeutic outcome on an individualized basis. However, and as a general rule, without correct study design, strategy and implementation of robust analytical methodologies, the efforts, efficiency and expectations to make biomarkers (especially phosphorylated kinases) a useful reality in the near future, can easily be hampered.

## Findings

### Overview

Proteomics and phosphoproteomics clinical research studies imply the comprehensive analysis of the proteins which are expressed in cells or tissues, and can be employed at different stages (*e.g*. healthy *vs*. disease). Therefore, comparative proteomics can distinguish small, but relevant changes in protein modifications in their structure -post-translational modifications (PTMs)- at a depth of several thousand proteins to facilitate drug target identification.

Chemical and Biochemical proteomics can be used to identify drug-target interactions and subsequently analyze drug specificity and selectivity. Furthermore, phosphoproteomic approaches can be exploited to monitor changes in phosphorylation events in order to characterize drug actions on cell signalling pathways and/or signalling cascades. In addition, functional proteomic approaches, can be employed to investigate protein-protein and protein-ligand interactions in order to: (i) improve the knowledge or the clarification of the mechanism of drug action, (ii) achieve relevant protein-identifications of disease-related sub-networks and (iii) reach the important step of innovation of novel drug targets.

Furthermore, proteins are currently the major drug targets, and therefore play a critical role in the process of modern drug design. This typically involves: (1) the construction of drug compounds based on the structure of a specific drug target, (2) validation for therapeutic efficacy of the drug compounds, (3) evaluation of drug toxicity, and finally, (4) clinical trial.

Finally, tissue imaging MS is being extended as a current promising technique for reproductive research. Advances in MS imaging will inevitably attract biologists and clinicians as the advantages and power of this technology become more widely known. We will detail, in a simple manner, relevant clues of current proteomic, phosphoproteomic and MS strategies and techniques useful for clinical advances [1].

#### Phosphoproteomics relevance in signalling transduction pathways

It is well known that phosphoproteomics and MS-based recent advancements have made these approaches the ideal way by which to study signal transduction although it implies high speciality and tedious research studies. In addition, individual protein phosphorylation events often have important roles and clues in broad signalling networks within a cell. Unfortunately, while phosphorylation of kinases frequently, mainly regulates their own activity, they are commonly under-represented in phosphoproteomic studies, partly due to their low expression within the cell. Nevertheless, a viable solution to this drawback has been successfully proven via kinase affinity purification techniques. Thus, important improvements are helping to achieve relevant data of phosphorylated kinases - those proteins being the "key" of signalling pathways and network- connectivity among different signalling cascades.

Phosphatases are playing equally important roles in regulating signalling pathways through the removal of phosphoryl groups from proteins. Indeed, depleting cells of specific protein phosphatases and employing phosphoproteomic approaches, can be used to determine which proteins are regulated by the phosphatase of interest, either directly or downstream [2-7].

The best studies of mitogen activated protein kinase (MAPKs) are the extracellular signal regulated protein kinases (ERK). ERKs phosphorylate cytoplasmic targets migrate to the nucleus where they can activate transcription factors involved in cellular proliferation. As a general view of the orchestrated signalling pathways, it is important to know that following the communication of the signal to different cellular compartments are (1) signal processing and (2) amplification by plasma membrane proximal events.

The activation of multiple signal cascades by (1) receptors, (2) different protein PTMs, (3) crosstalk between signalling pathways and (4) feedback loops to ensure optimal signalling output, are involved in this process. Also, the binding of receptor Tyrosine (Tyr) kinases (RTKs) to their cognate ligands at the cell surface results in receptor dimerization and autophosphorylation. Phosphorylated Tyr residues subsequently serve as docking sites to recruit signalling mediators, such as growth factor receptor-bound protein 2 (GRB2).

Multiple signalling cascades such as (1) the phosphoinositide-3 kinase (PI3K)-AKT, (2) Ras-Raf- extracellular signal-regulated kinase (ERK) mitogen-activated protein kinase (MAPK), and (3) signal transducer and activator of transcription (STAT) pathways are activated by the assembly of these signalling complexes. On the other hand, (4) Casitas B-lineage lymphoma (CBL)-mediated ubiquitylation of RTKs controls their endocytosis and the duration of receptor signalling. In addition, binding of tumour necrosis factor-α (TNFα) to its receptor, TNFR1, induces trimerization of the receptor and recruitment of the adaptor protein TNFR1-associated death domain (TRADD). These functions as a hub to assemble a multi-protein signalling complex containing TNFR-associated factor 2 (TRAF2), receptor interacting Ser/Thr protein kinase 1 (RIPK1) and nuclear factor-κB (NF-κB) essential modulator (NEMO). The result is the activation of different signalling networks, such as the ERK MAPK, p38 MAPK and NF-κB pathways. Proteins in the MAPK signalling pathways are activated by both RTKs and TNFα, which allows cells to integrate multiple signals [8-20].

#### Advantages/disadvantages and clues of most used MS-based tools for the detection of phosphorylated proteins/peptides

Several analytical techniques exist for the analysis of phosphorylation, *e.g*., Edman sequencing and ^32^P-phosphopeptide mapping for localization of phosphorylation sites, but these methods do not allow high-throughput analysis or imply very laborious operations [21], while using MS, high-throughput analysis of phosphorylated protein residues can be developed [22,23]. On the other hand, phosphospecific antibodies are routinely used to immunoprecipitate and therefore to enrich in phosphorylated proteins from complex mixtures [24], but, currently, there are no antibodies available commercially suitable for enriching all proteins that are phosphorylated, and thus, these proteins must be purified or enriched from complex mixtures using alternative methods [25].

When carrying out in-gel or in-solution trypsin digestion of protein complex mixtures, the resulting phosphopeptides and non-phosphopeptides can be loaded into different metal ion chromatographies (*e.g*. Immobilized Metal ion Affinity Chromatography IMAC (Fe^3+^), and Titanium Dioxide TiO_2 _[26]) in order to enrich in phosphopeptides. The enriched solution can also be submitted into different reverse-phase chromatographies (*e.g*. Graphite powder [27], POROS R3) [25] in order to clean and desalt those phosphopeptides previously eluted. Moreover, all these types of chromatographies will reduce the suppression of phosphorylated peptides in the mass spectra.

Using IMAC (Fe^3+^) and also (TiO_2_) [26], the negatively charged phosphopeptides are purified by their affinity to positively charged metal ions, but some of these methods suffer the problem of binding acidic, non-phosphorylated peptides. Ficarro and co-workers [22], circumvented this problem on IMAC (Fe^3+^) by converting acidic peptides to methyl esters but increasing the spectra complexity and requiring lyophilization of the sample, which causes adsorptive losses of especially phosphopeptides [28]. Ficarro *et al.*, were able to sequence hundreds of phosphopeptides from yeast, including Slt2p kinase, but the level of phosphorylated residues identified from kinases were low compared to the ones from phosphoproteins highly expressed within the cell. Fairly recently, TiO_2 _chromatography using 2,5-dihydroxybenzoic acid (DHB) was introduced as a promising strategy by Larsen *et al. *[26],. TiO_2_/DHB resulted in higher specificity and yield as compared to IMAC (Fe^3+^) for the selective enrichment of phosphorylated peptides from model proteins (*e.g*. lactoglobulin bovine, casein bovine, etc).

Another important limitation concerning the phosphoenrichment methods is that mainly phosphopeptides from highly expressed proteins within cells can be purified, while the ones from phosphorylated proteins with low level expression (*e.g*. kinases) do not bind so well to those resins. This is due to the low proportion of this kind of proteins, or on the other hand, their available amount binds to metal ions although it is not sufficient to be detected by MS. The combination of Strong Cation Exchange Chromatography (SCX) with IMAC (Fe^3+^) has been proven on yeast, resulting in a huge number of phosphorylated residues identified (over 700, including Fus3p kinase) [23]. Although more than 100 signalling proteins and functional phosphorylation sites were identified, including receptors, kinases and transcription factors, it was clear that only a fraction of the phosphoproteome was revealed [23].

It is evident that methodologies to enrich for phosphorylated residues from kinases should be improved. However, this is not straightforward for several reasons: (a) the low abundance of those signalling molecules within cells, and (b) the stress/stimulation time-duration, as only a small fraction of phosphorylated kinases are available at any given time as a result of a stimulus. Also, the time adaptation over signalling pathways is a relevant and fast factor for kinases phosphorylation [29], and (c) the current phosphoenrichment methods, which are mainly successful to purify phosphopeptides from highly expressed proteins.

In a simple manner, we will detail the manual validation of the phospho-data (assignments of the phosphate group on specific amino acids) obtained in an MS experiment during CID (Collision Induced Dissociation) operations. When peptide ions are fragmented via CID, series of *y*- and *b*- ions are formed [30,31]. The peptide sequence is obtained by correlating mass difference between peaks in the *y*-ion series or between peaks in the *b*-ion series with amino acid residue masses. The CID fragmentation mainly occurs on the peptide backbone, and sequence information is obtained. In relation to phosphotyrosine residues, partial neutral loss is observed (HPO_3_, 80 m/z) in MS2 mode, and the phosphate group on tyrosine (Tyr) residues is more stable than on serine (Ser) and threonine (Thr) residues. Also, the phospho-finger-print characteristic of phosphotyrosine is the phosphotyrosine immonium ion (~216 Da) [32,33]. Via MS3 mode, the ion originating from neutral loss (NL) of phosphoric acid (H_3_PO_4_) can be selected for further fragmentation. Then, the selected ion is automatically selected for further fragmentation after neutral loss fragmentation. Therefore, it is possible to add extra energy for the fragmentation of peptide backbone.

Nevertheless, the MS3 mode requires that the phosphorylation on Ser and Thr residues are labile and conventional fragmentation via CID commonly resulting in the partial NL of H_3_PO_4_, (98 m/z) in MS2 mode. This is due to the gas phase β-elimination of the phosphor-ester bond and thus, dehydroalanine (Ser ~69 Da) and dehydro-2-aminobutyric acid (Thr ~83 Da) are generated [32,33].

In addition, as alternative phosphopeptide enrichment strategies, phosphopeptides can be de-protected and collected under acidic conditions and a variety of chemical methodologies have likewise appeared. BEMA (β-elimination/Michael addition), takes advantage of the ease of β-elimination of phosphorylated Ser and Thr residues at basic pH and the ability to subject the resulting dehydroalanine/methyl-dehydroalanine products to Michael addition with a desired tag for affinity purification [34-36]. In addition, Calcium phosphate precipitation (CPP) has been proven to be a fast, economical, and simple enrichment technique [37] in exchange for diminished specificity. Moreover, PhosphorAmidate Chemistry (PAC) is another important approach in which phosphopeptides are coupled to a solid-phase support such as an amino-derivatized dendrimer or controlled-pore glass derivatized with maleimide for selection [38,39].

#### Tandem MS Methodology -basic issues useful for Phosphoproteomics via ElectroSpray Ionization (ESI)

It can be taken as a general rule, that during MS-based experiments, a phosphopeptide mixture is separated using capillary liquid chromatography (LC). A typical separation column is 25 to 100 microns in diameter and 5 to 30 cm in length. The eluent is concurrently introduced into the mass spectrometer *via *electrospray ionization (ESI). ESI is a process that generates multiply protonated gas-phase peptide cations. The mass-to-charge ratio (*m/z*) and intensity (*I*) of the intact peptide precursors are recorded by an initial MS scan - commonly referred to as a full scan MS. Then, *m/z *values for peaks (list of masses) with high intensity are automatically selected in order of decreasing abundance for sequencing by tandem MS (MS/MS). This process of precursor selection, dissociation, and fragment ion mass analysis is repetitively performed on analyte species as they elute from the LC column. Ideally, MS/MS interrogation of a phosphorylated peptide generates a series of fragment ions that differ in mass by a single amino acid, so that the peptide primary sequence and position of the phosphorylated modifications can be determined. This necessitates peptide bond cleavage that is not only specific to the peptide backbone, but is robust enough to elucidate differences in peptides whose primary amino acid sequence are the same, yet vary in the site of phosphorylation (*e.g*., positional isomers) [40]

The dominant NL peak in the fragmentation spectra of phosphopeptides obtained via traditional collisionally induced dissociation (CID) has received much attention [41-43]. The NL peak can easily suppress sequence diagnostic ion peaks causing identification of the peptide to become extremely difficult and sometimes impossible.

Since the use of ion traps, currently, as the most common mass spectrometers of performing phosphoproteome analyses, there have been various attempts to combat this specific problem. Modified fragmentation regimes have been introduced, such as (a) NL triggered MS3 or (b) multistage activation (MSA), which alleviate the neutral loss issue. NL MS3 and MSA methods allow fragmenting of the NL peak of the precursor ion further, in order to generate more backbone cleavages. These "extra" generated backbone cleavages, then form more diagnostic sources for peptide sequencing [23,44-46].

**Figure 1:**
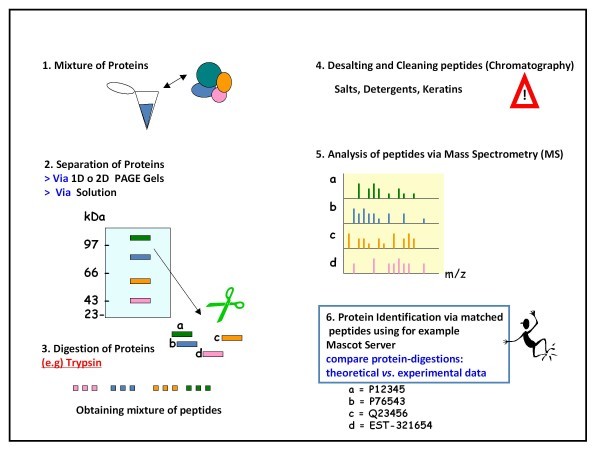
**Identifying proteins via Proteomics-Mass Spectrometry**. The mixture of proteins (or just one protein) must be digested to obtain peptides. The resulting peptides have to be cleaned and desalted via chromatography (*e.g*. POROS R2) to avoid salts and detergents, which artefact the MS analysis. Subsequently, the desalted and cleaned peptides are injected into the mass spectrometer. Finally, the matched peptides allow the identification of the proteins using databases (*e.g*. Mascot Server).

**Figure 2:**
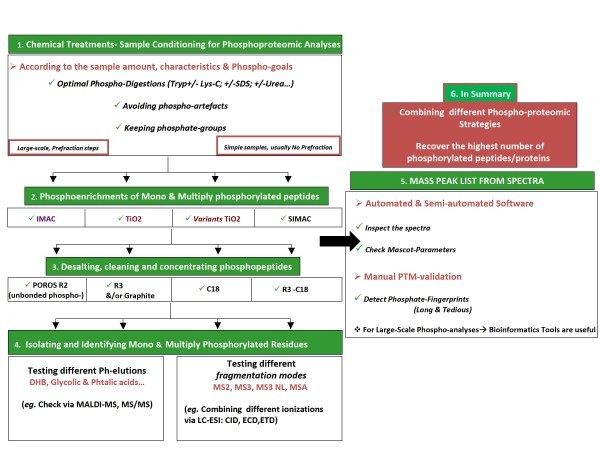
**Flow-through of Current Phospho-proteomic Analysis**. Using phosphoenrichments (*e.g*. IMAC, TiO_2_; SIMAC) we are capable of isolating phosphorylated peptides and discard un-phosphopeptides. The isolated phosphopeptides have to be cleaned and desalted via chromatography (*e.g*. POROS R3, Disks C18 or graphite-, which isolate hydrophilic peptides) before the MS analysis. Finally, the desalted and cleaned phosphopeptides are injected into the mass spectrometer. Different types of ionization can be used (*e.g*. Matrix-Assisted Laser Desorption/Ionization MALDI or ElectroSpray Ionization ESI). Also, different kinds of fragmentations can be used (*e.g*. CID, ETD, ECD). In addition, different MS modes can be useful, for example: MS/MS, MSA, MS3NL. As a general rule, positive MS mode is currently more efficient than negative mode for phosphoproteomic studies. It is always necessary to test and combine different phosphoenrichments together with different MS strategies to recover and identify the maximum level of phosphopeptides. This will imply a high efficiency for your clinical research study. The resulting data (phosphorylated proteins identified) must be coupled to bioinformatic tools (software) in order to improve the biological understanding.

Alternatively, Electron transfer dissociation (ETD) and electron capture dissociation (ECD) have also shown great promise since the phosphate group remains attached during and after activation. Many detected phosphopeptides contain multiple Ser/Thr/Tyr (serine, threonine, and tyrosine) residues representing the likelihood that there is more than one possible location for the site of phosphorylation within the peptide. The abundant NL observed in low energy CID can hamper the correct assignment of the phosphor-sites in such peptides. Thus, a concerted effort has been made to understand, in detail, the rules of phosphopeptide fragmentation [47-51]. Figures 1 and 2 illustrate the flow-through to identify proteins via proteomics-MS, and different phosphoproteomic strategies to ensure high efficiency for clinical research study, respectively.

#### Discovering Biomarkers via OMICS Tools

MS-based proteomics technologies are capable of identifying hundreds to thousands of proteins in cells, tissues, and biofluids. Proteomics may, therefore, provide the opportunity to elucidate new biomarkers and pathways without a prior known association with a specific disease. However, important obstacles remain.

Additionally, improved biomarkers are of vital importance for cancer detection, diagnosis and prognosis. Significant advances in understanding the molecular basis of disease are being made in genomics, while proteomics will ultimately delineate the functional units of a cell: proteins and their intricate interaction networks and signalling pathways in health and disease. Much progress has been made to characterize thousands of proteins qualitatively and quantitatively in complex biological systems by use of multi-dimensional sample fractionation strategies, MS and protein micro-arrays. Comparative/quantitative analysis of high-quality clinical biospecimen (*e.g*., tissue and biofluids) of the human cancer proteome landscape can potentially reveal protein/peptide biomarkers responsible for this disease by means of their altered levels of expression, PTMs as well as different forms of protein variants. Despite technological advances in proteomics, major hurdles still exist at every step of the biomarker development pipeline [52-63].

The field of proteomics, in the post-genome era, incited great interest in the pursuit of protein/peptide biomarker discovery especially since MS demonstrated the capability of characterizing a large number of proteins and their PTMs in complex biological systems, in some instances even quantitatively. Technological advances, such as protein/antibody chips, depletion of multiple high abundance proteins by affinity columns, and affinity enrichment of targeted protein analytes, as well as multidimensional chromatographic fractionation, have all expanded the dynamic range of detection for low abundance proteins by several orders of magnitude in serum or plasma, making it possible to detect the more abundant disease-relevant proteins in these complex biological matrices [63-71]. Nevertheless, plasma and cell-extract based discovery research studies aimed at identifying low abundance proteins (*e.g*. some kinases) are extremely difficult. Therefore, it is necessary to develop significant technological improvements related to identifying these low abundance, yet high biological impact molecules. Furthermore, if these protein kinases to be studied contain PTMs, it is important to know that spatial and temporal factors can decrease the efficiency of our study (*e.g*. many kinases are regulated by phosphorylation of the activation loop, which then directly reflects cellular kinase activity).

Moreover, proteomics has been widely applied in various areas of science, ranging from the deciphering of molecular pathogenesis of diseases, the characterization of novel drug targets, to the discovery of potential diagnostic and prognostic biomarkers, where technology is capable of identifying and quantifying proteins associated with a particular disease by means of their altered levels of expression [72-74] and/or PTMs [75-77] between the control and disease states (*e.g*., biomarker candidates). This type of comparative (semi-quantitative) analysis enables correlations to be drawn between the range of proteins, their variations and modifications produced by a cell, tissue and biofluids and the initiation, progression, therapeutic monitoring or remission of a disease state.

PTMs including phosphorylation, glycosylation, acetylation and oxidation, in particular, have been of great interest in this field as they have been demonstrated as being linked to disease pathology and are useful targets for therapeutics.

In addition to MS-based large-scale protein and peptide sequencing, other innovative approaches including self-assembling protein microarrays [78] and bead-based flow cytometry [79,80] to identify and quantify proteins and protein-protein interaction in a high throughput manner, have furthered our understanding of the molecular mechanisms involved in diseases.

#### Utilities of Matrix-assisted laser desorption/ionization tissue imaging MS

Matrix-assisted laser desorption/ionization (MALDI) tissue imaging mass spectrometry is particularly promising among the numerous applications of mass spectrometry. It is used for testing and analyzing the spatial arrangement of a wide range of molecules including proteins, peptides, lipids, drugs and metabolites, directly in thin slices of tissue. In the field of proteomics, the technology avoids tedious and time-consuming extraction and fractionation steps classically required for sample analysis. Furthermore, MALDI imaging MS is increasingly recognized as a powerful method for clinical proteomics, particularly in cancer research. This recent technology has particular potential for the discovery of new tissue biomarker candidates, for classification of tumors, early diagnosis or prognosis, elucidating pathogenesis pathways and therapy monitoring. Over recent years, MALDI imaging MS has been used for molecular profiling and imaging directly in male and female reproductive tissues.

In summary, the wealth of advances in MS imaging will inevitably attract experts in OMICS (e.g. genomics, proteomics, bioinformatics) and clinicians, as the advantages and power of this technology become more widely known. In addition, it is important to point out for efficient clinical studies, that the identification of protein biomarkers in easily accessible biological fluids has potential for the development of minimally invasive procedures for early diagnostics, but the analysis of body fluids such as plasma, serum and urine is complicated by their wide dynamic range of protein expression, the variation in their composition and their sensitivity to sample handling [81-83].

#### Concluding remarks and future needs

Phosphoproteomics is a branch of proteomics that identifies, catalogs, and characterizes proteins containing a phosphate group as a PTM. Furthermore, phosphoproteomics provides clues on which protein or pathway might be activated because a change in phosphorylation status almost always reflects a change in protein activity. Indeed, it can indicate which proteins might be potential drug targets as exemplified by the kinase inhibitor. While phosphoproteomics will greatly expand knowledge about the numbers and types of phosphoproteins, its greatest promise is the rapid analysis of entire phosphorylation based signalling networks. Nevertheless, methodologies to enrich for phosphorylated residues from kinases should be improved, especially due to their low abundance of those signalling molecules within cells.

To summarize, clinical proteomics-MS has come a long way in the past decade in terms of technology/platform development, protein chemistry, and together with bioinformatics and other OMICS tools to identify molecular signatures of diseases based on protein pathways and signalling cascades. Hence, there is great promise for disease diagnosis, prognosis, and prediction of therapeutic outcome on an individualized basis. In addition, imaging MS will have a major impact in reproductive research by opening new avenues to the understanding of various molecular mechanisms and the diagnosis of reproductive pathologies. However, and as a general rule, without correct study design, strategy and implementation of robust analytical methodologies, the efforts, efficiency and expectations to make biomarkers (especially phosphorylated kinases) a useful reality in the near future, can easily be hampered.

## Competing interests

The authors declare that they have no competing interests.

## Authors' contributions

Authors EL SRM, JLP and LM carried out Clinical Proteomics-MS studies for this short-review, in order to develop future Clinical Proteomic-Phosphoproteomic MS research studies and publish this article. All authors read and approved the final manuscript.

## Author information

EL PhD Scientist, -Hospital Universitario 12 de Octubre-, was a recipient of a Postdoc fellowship "Ministerio de Ciencia e Innovacion de España". And coordinates a Multidisciplinary Project "Red de Hospitales Nacionales de España" for Clinical OMICS Research, -Hospital Universitario Infantil Niño Jesús-.

SRM and JLP are MD PhD and hold a tenured position at Hospital Universitario 12 de Octubre.

LM is Professor at Universidad Autónoma Medicina de Madrid, and holds a tenured position at Hospital Universitario Infantil Niño Jesús.
